# Metabolic Imbalance Effect on Retinal Müller Glial Cells Reprogramming Capacity: Involvement of Histone Deacetylase SIRT6

**DOI:** 10.3389/fgene.2021.769723

**Published:** 2021-11-04

**Authors:** L Francisco Sanhueza Salas, Alfredo García-Venzor, Natalia Beltramone, Claudia Capurro, Debra Toiber, Dafne Magalí Silberman

**Affiliations:** ^1^ Centro de Estudios Farmacológicos y Botánicos (CEFYBO-UBA-CONICET), Facultad de Medicina, Universidad de Buenos Aires, Buenos Aires, Argentina; ^2^ Department of Life Sciences, Ben-Gurion University of the Negev, Beer Sheva, Israel; ^3^ The Zlotowski Center for Neuroscience, Ben-Gurion University of the Negev, Beer Sheva, Israel; ^4^ Departamento de Ciencias Fisiológicas, Laboratorio de Biomembranas, Instituto de Fisiología y Biofísica “Bernardo Houssay” (IFIBIO‐HOUSSAY), Consejo Nacional de Investigaciones Científicas y Técnicas (CONICET), Universidad de Buenos Aires, Buenos Aires, Argentina

**Keywords:** retina, müller cells, metabolism, reprogramming, SIRT6

## Abstract

Retinal Müller glial cells (MGs) are among the first to demonstrate metabolic changes during retinal disease and are a potential source of regenerative cells. In response to a harmful stimulus, they can dedifferentiate acquiring neural stem cells properties, proliferate and migrate to the damaged retinal layer and differentiate into lost neurons. However, it is not yet known how this reprogramming process is regulated in mammals. Since glucose and oxygen are important regulatory elements that may help directing stem cell fate, we aimed to study the effect of glucose variations and oxidative stress in Müller cells reprogramming capacity and analyze the participation the histone deacetylase SIRT6, as an epigenetic modulator of this process. We found that the combination of high glucose and oxidative stress induced a decrease in the levels of the marker glutamine synthetase, and an increase in the migration capacity of the cells suggesting that these experimental conditions could induce some degree of dedifferentiation and favor the migration ability. High glucose induced an increase in the levels of the pluripotent factor SOX9 and a decrease in SIRT6 levels accompanied by the increase in the acetylation levels of H3K9. Inhibiting SIRT6 expression by siRNA rendered an increase in SOX9 levels. We also determined SOX9 levels in retinas from mice with a conditional deletion of SIRT6 in the CNS. To further understand the mechanisms that regulate MGs response under metabolic impaired conditions, we evaluated the gene expression profile and performed Gene Ontology enrichment analysis of Müller cells from a murine model of Diabetes. We found several differentially expressed genes and observed that the transcriptomic change involved the enrichment of genes associated with glucose metabolism, cell migration, development and pluripotency. We found that many functional categories affected in cells of diabetic animals were directly related to SIRT6 function. Transcription factors enrichment analysis allowed us to predict several factors, including SOX9, that may be involved in the modulation of the differential expression program observed in diabetic MGs. Our results underline the heterogeneity of Müller cells response and the challenge that the study of metabolic impairment *in vivo* represents.

## Introduction

In mammals, disease or injury of the retina leads to irreparable vision loss. Conversely, some teleost fish mount a regenerative response to retinal injury that is capable of restoring lost sight (Lindsey and Powers, 2007; Sherpa et al., 2008). Müller glial cells (MGs), the main glial cells of the retina, are key to successful regeneration. In response to a harmful stimulus they can rapidly dedifferentiate acquiring neural stem cells properties, proliferate and migrate to the damaged retinal layer and differentiate into lost neurons (Fischer and Reh, 2003; Ooto et al., 2004). In mammals, MGs generally respond to injury by reactive gliosis accompanied by hypertrophy, but they rarely re-enter the cell cycle and regenerate new neurons (Bringmann et al., 2009; Burda and Sofroniew, 2014). These data suggest that the ability of MGs to dedifferentiate in response to retinal injury would be a crucial difference between the regenerative responses of fish and mammals (Ramachandran et al., 2010). In this sense, it is not yet known how to unlock the neurogenic potential of the mammalian MGs.

As part of the central nervous system, the retina shares the characteristic high metabolism of the brain. It is currently accepted that many retinopathies like age-related macular degeneration (Zhang et al., 2020), diabetic retinopathy (Liew et al., 2017) and retinitis pigmentosa (Fu et al., 2019) could be associated with both systemic and tissular metabolic impairment. Müller cells play a critical role in retinal metabolism and are among the first cells to demonstrate metabolic changes in retinal stress or disease (Bringmann et al., 2009). Reactive gliosis of MGs is characterized by non-specific and specific responses. These responses involve changes in their morphology, biochemistry and physiology (Bringmann et al., 2009). Early after injury, gliosis plays a neuroprotective role releasing neurotrophic factors and antioxidants. However, if gliosis persists, the dedifferentiation of cells contributes to neuronal cell death, e.g., *via* the impairment of neurotransmitter removal promoting excitotoxicity (Newman, 2004). Generally, an impairment of the supportive functions of Müller cells may have an additive effect on dysfunction and neuron loss by increasing the susceptibility of neurons to stressful stimuli in the diseased retina. Thus, and considering that many metabolic diseases are often accompanied by neuronal dysfunction, a proper understanding of the mechanisms implicated in Müller cells under metabolic impaired conditions is essential for the development of efficient therapeutic strategies.

Somatic reprogramming and pluripotency are characterized by a transition from oxidative metabolism into anaerobic glycolysis (Folmes et al., 2011). Interestingly, the NAD^+^-dependent histone deacetylase SIRT6 has been shown to act as a key modulator of glucose homeostasis and its absence, or inactivation, favors the switch to anaerobic glycolysis by the upregulation of the levels of the glucose transporter GLUT1 and of several glycolytic genes (Zhong et al., 2010). Furthermore, SIRT6 was described as a direct regulator of the expression of several core pluripotent genes by the dependent deacetylation of acH3K56 and acH3K9 during embryonic stem cells (ESC) differentiation (Etchegaray et al., 2015), further suggesting a key role of SIRT6 as a regulator of these cells’ pluripotency capacity. Moreover, we have recently demonstrated that SIRT6 would regulate early neurodegenerative events observed during Diabetic Retinopathy development by modulating the expression of key neural factors in Müller cells (Zorrilla-Zubilete et al., 2018). In this work we aimed to study the effect of glucose variations and oxidative stress in Müller cells’ reprogramming capacity and analyze the participation of SIRT6 as a potential epigenetic modulator of this process.

For this purpose, Müller glial cells were cultured with different glucose concentrations and under oxidative stress conditions. We analyzed the levels of the glial cell marker glutamine synthetase (GS) and the reprogramming factor SOX9. Additionally, we tested MGs migration capacity under these stimuli. We studied the involvement of SIRT6 in the reprogramming capacity by analyzing SIRT6 levels and the acetylation status of one of its substrates (acH3K9). We also evaluated the levels of SOX9 in siRNA SIRT6-silenced cells. We also determined the levels of SOX9 in retinas from mice with a conditional deletion of SIRT6 in the CNS (Nes-Cre^−/−^) and performed gene and transcription factor enrichment analysis of retinal Müller cells from a murine model of Diabetes. We found that the combination of high glucose (HG) concentrations and oxidative stress may induce some degree of dedifferentiation of MGs and favors their migration ability. We also observed that HG induced an increase in the levels of the well-known pluripotent factor SOX9 and a decrease in SIRT6 levels accompanied by the increase in the acetylation levels of H3K9. Notably, inhibiting SIRT6 expression in MGs by siRNA rendered an increase in SOX9 levels. We noticed a moderate, though not significant, increment in SOX9 levels in retinas from Nes-Cre^−/−^ mice suggesting that *in vivo* modulation of this transcription factor by SIRT6 would be indirect and may involve other players. Gene ontology (GO) enrichment analysis showed differentially expressed genes in categories related to metabolism, cell migration and pluripotency in MGs from diabetic retinas. Interestingly, we found that many functional categories affected in cells from these animals were directly related to SIRT6 function like glycolysis, Hif1α pathway and phototransduction. Moreover, we identified 67 predicted transcription factors, including SOX9, that could be responsible for the transcriptional changes observed in diabetic MGs. The GO enrichment analysis of these TFs showed that these genes are enriched in biological processes linked to cellular and brain development as well as neurogenesis and pluripotency. The diversity of Müller cells’ response to a metabolic defy represents a major challenge in the understanding of the regulation of reprogramming processes *in vivo*.

## Methodology

### Cell Cultures

The spontaneously immortalized retinal Müller glial cell line MIO-M1 (kindly provided to CC by Dr. Astrid Limb, University College London, London, United Kingdom) was grown following a modified protocol adapted from the previously described (Netti et al., 2017). Monolayers of cells were cultured in the presence of Dulbecco’s Modified Eagle Medium (DMEM) either with high (25 mM) or low glucose (5 mM) concentrations, glutamax (GibcoTM) supplemented with 10% fetal calf serum (FCS) containing 5 µg/ml streptomycin and 5 U/ml penicillin at 37°C in a humidified atmosphere with 5% CO_2_. Cells were routinely subcultured every week.

Different concentrations of hydrogen peroxide (H_2_O_2_) were tested (50, 100 and 500 µM) in order to find the optimal dose that generates oxidative damage without affecting cell viability (Supplementary Figure S1). According to our observations, 100 µM of H_2_O_2_ were used to induce oxidative damage and were added to the cultures 2 h prior to determinations unless otherwise specified.

### Immunofluorescence

For cultures, cells were fixed with 4% paraformaldehyde for 10 min, permeabilized with 0.1% Triton X-100 in 0.1 M PBS for 10 min, and blocked with 5% normal horse serum for 1 h at 18–22°C. After overnight incubation at 4°C with the corresponding primary antibodies [anti-SOX9 (Abam, ab5535), γH2AX (Abcam, Cat# ab2893), anti-acetyl H3K9 antibody (dil 1: 500; Abcam), anti-glutamine synthetase (1 : 250; Millipore Corporation, Bedford, MA, United States)], cells were incubated with the secondary antibody for 1 h at 18–22°C (Alexa Fluor^®^ 555 Goat Anti-Mouse IgG, 1 : 500, Alexa Fluor^®^ 488 Goat Anti-rabbit IgG, 1 : 500). Some wells were left untreated (no primary antibodies) as a control. 40,6-diamidino-2-phenylindole (DAPI) was used after immunostaining to dye the nuclei and a Nikon Eclipse (E300) fluorescence microscope was used to visualize the marks.

For retinas, paraffin-embedded tissues were sectioned and treated as previously described (Fernandez et al., 2013). After deparaffinization, cross sections were immersed in 0.1% Triton X-100 in 0.1 M PBS for 10 min and antigen retrieval was performed by heating at 90°C for 30 min in citrate buffer (pH 6.3). Sections were blocked and incubated overnight at 4°C with the corresponding primary antibodies as described for cells.

### Western Blot

Western blot analysis was carried out as previously described (Silberman et al., 2014). Briefly, for histone-enriched extracts, cells or retinas were homogenized in lysis buffer (10 mM Hepes pH 7.4, 10 mM KCl, 0.05% NP-40) containing protease inhibitors (Roche Molecular Biochemicals, Indianapolis, IN, United States). Homogenates were kept in ice for 20 min and centrifuged at 22,000 g for 10 min at 4°C. Supernatant was used for cytoplasmic protein determination and pellets were resuspended in 2–5 volumes of HCl 0.2 N and kept for 20 min on ice followed by centrifugation at 22,000 g for 10 min at 4°C. Supernatants were neutralized with 1 M TRIS-Cl pH 8 and an aliquot was used to determine protein concentration. 4–20% gradient Tris glycine sodium dodecyl sulfate-polyacrylamide electrophoresis pre-casted gel (Bio-Rad Laboratories, Hercules, CA, United States) was used to separate samples (10–30 μg protein/well) that were transferred to polyvinylidene difluoride and incubated with the corresponding primary antibodies overnight at 4°C followed by secondary antibodies incubation for 1 h at room temperature. Bands were visualized by enzymatic chemiluminiscence (ECL, Western Blotting Analysis System, Amersham Biosciences, Buenos Aires, Argentina). Developed membranes were scanned and the intensity of bands was determined by using the ImageJ program (National Institutes of Health, Bethesda, MD, United States).The antibodies used were as follows: anti- SIRT6 (Abcam, Cat# ab62739), anti-AcH3K9 (Abcam, Cat# ab12179, RRID:AB_298910), anti- Histone 3 (Abcam, Cat# ab39655, RRID:AB_732921), anti-β-actin (Sigma-Aldrich, Cat# A5316, RRID:AB_476,743), anti-Glutamine synthetase (Sigma, Cat# G2781), anti-SOX9 (Abam, Cat# ab5535), gH2AX (Abcam, Cat# ab2893).

### SIRT6 siRNA Silencing

SIRT6 siRNA (Sigma-Aldrich, #EMU02471) or siRNA Universal Negative control (Sigma-Aldrich, #SIC001) were used for silencing experiments. Cells were transiently transfected using Lipofectamine 2000 according to manufacturer’s instructions. Cells were collected 48 h post-transfection for determinations.

### Conditional Deletion of SIRT6 in the CNS

All animal procedures were in strict accordance with the Association for Research in Vision and Ophthalmology (ARVO) Statement for the Use of Animals in Ophthalmic and Vision Research. The conditional deletion of SIRT6 in the CNS was achieved by inserting a Neo cassette (flanked by two Frt sequences) together with Sirt6 exon 2 flanked by two loxP sites. The targeted ES cells (V6.5) were injected into C57BL6/J blastocysts to generate chimeric mice. By crossing the chimeras with a mouse expressing the Flpe endonuclease, the Neo cassette was deleted *in vivo* and the resulting mice were backcrossed for three generations with C57BL6/J mice (Jackson Laboratories, stock nr. 000664, Bar Harbor, ME, United States) to obtain heterozygous mice that were 97% C57BL6/J background. Mice were interbred to obtain homozygous Sirt6 fl/fl mice that were crossed with C57BL/Nestin-Cre/J mice (Jackson Laboratories, stock nr. 003771) resulting in mice expressing the Cre recombinase under Nestin promoter. Cre recombinase exposure leads to exon 2 excisions and the appearance of a premature stop codon. Both female and male mice were used.

### q-PCR

RNeasy Mini Kit (Qiagen, Valencia, CA, United States) was used to isolate total RNA and cDNA was generated using QuantiTect Reverse Transcription Kit (Qiagen) according to manufacturer’s instructions. qPCR was carried out using Brilliant SYBR Green qPCR Master Mix Kit (Stratagene, La Jolla, CA, United States). Data were calculated using the ΔCt method. Primers used were as follow: Sirt6-F GGG​AAC​TTG​AAG​GAA​CCA​CA, Sirt6-R AGCCTGGGC TATAGCAGTGA, β-actin-F ACT​ATT​GGC​AAC​GAG​CGG​TTC, β-actin-R AAG​GAA​GGC​TGG​AAA​AGA​GCC.

### Wound Healing Assay

Cell migration was assessed by the wound healing assay as previously described (Rehman et al., 2019). Briefly, cells were seeded on 8 chamber culture slides (Falcon, Corning, REF354108) and when they reached 90% confluency the medium was replaced using 5% serum concentrations to limit the study to the assessment of the migration capacity minimizing the contribution of cell proliferation to gap filling. A scratch was manually made using a pipette tip and bright-field images were taken at different times using an Olympus IMT-2 microscope. Experiments were performed in triplicates. Wound contraction was quantified as the area of the gap between the lesion boundaries immediately after the wounding procedure (t0) and after 18 h (t18) using the ImageJ software.

### Enrichment Analysis, Data Collection and Processing

The published transcriptional profile dataset GSE1979 (Gerhardinger et al., 2005) generated by using retinal Müller cells from diabetic rats compared to cells from healthy controls was used to determine differentially expressed genes through the limma software (Ritchie et al., 2015). The list of differentially expressed genes is shown in Supplementary Table S1. The differentially expressed genes were selected based on a *p* value lower than 0.05, and a fold change above 1.2, without taking into account the FDR value. Gene Onthology enrichment analysis was done by using the algorithm Panther 16.0 (Mi et al., 2019; Mi et al., 2013) (Supplementary Table S2). The gene sets obtained were analyzed using the tool package of ShinyGO v0.60 Gene Ontology Enrichment Analysis (Ge et al., 2020) or Reactome database (Jassal et al., 2020) to get enriched GO terms and functional categories from the differential expressed genes dataset. Enrichment analysis was performed based on hypergeometric distribution followed by FDR correction; *p* value cut off 0.5.

### Transcription Factor Enrichment Analysis

We used the ChEA algorithm (Lachmann et al., 2010), a curated dataset that includes the ENCODE ChIP results in its analysis, and the Opossum 3.0 algorithm (Kwon et al., 2012), which primarily uses JASPER algorithm to predict TF binding sites. The list of enriched transcription factors from both algorithms were compared using a Venn diagram tool and the shared list was used to GO enrichment analysis.

### Statistics

Normality of data was assessed by the Shapiro-Wilk’s test prior to the analysis. Quantitative data were expressed as means ± SE of values obtained from the “n” described in each figure or from three independent experiments. Student’s t-test was used and differences were considered significant at *p* < 0.05. One way ANOVA followed by Bonferroni’s test was applied when multiple comparisons were required.

## Results

**FIGURE 1:**
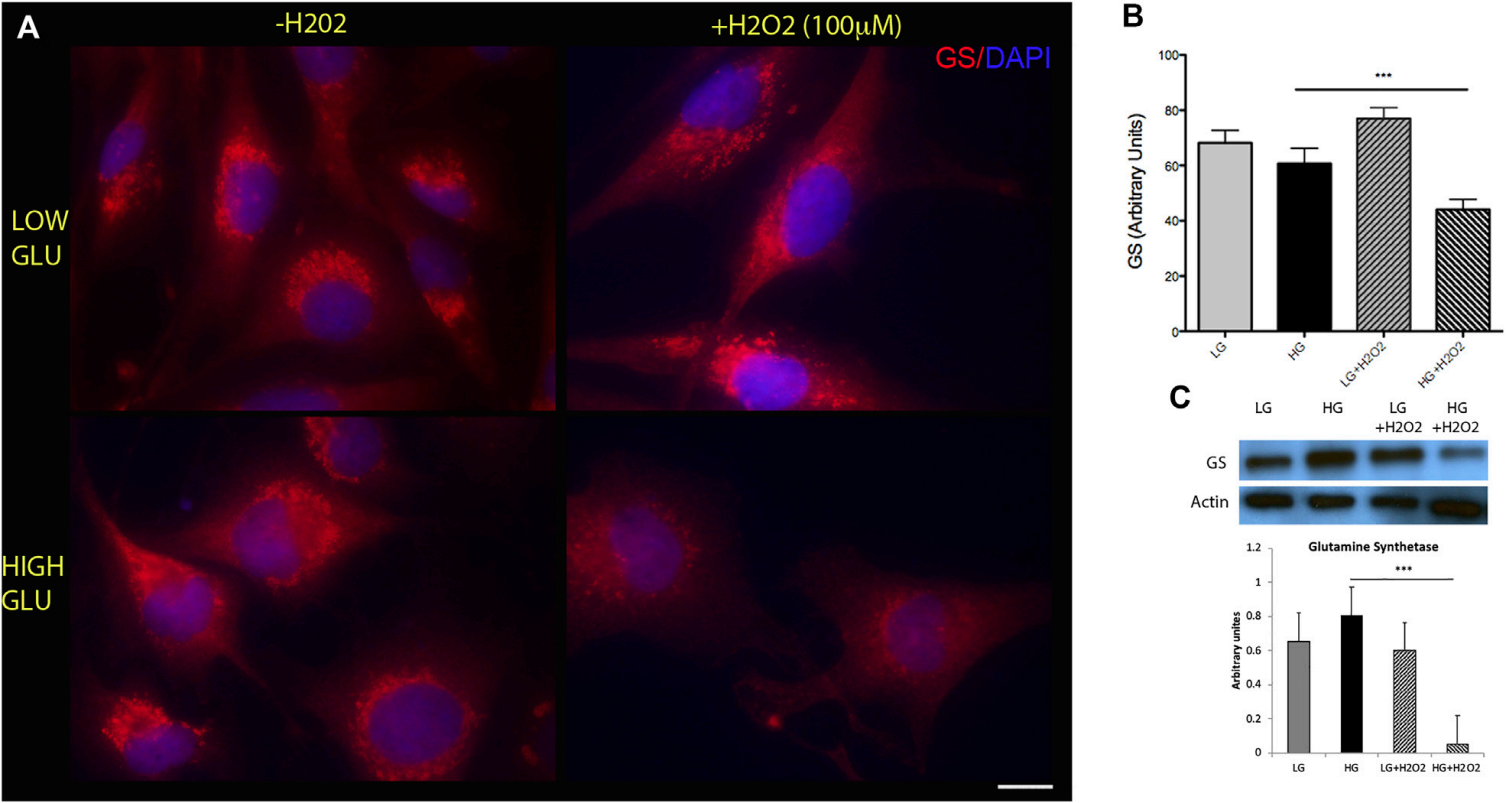
High glucose and oxidative stress induce a decrease in glutamine synthetase (GS) levels. **(A)** Representative immunofluorescence showing GS levels (red) in MGs cultured under low glucose (LG, 5 mM) or high glucose (HG, 25 mM) concentrations with (+H_2_O_2_, 100 μM) or without (−H_2_O_2_). DAPI was used for nuclei staining. Scale bar: 10 μm. **(B)** Quantification was performed using the ImageJ program and differences were analyzed using the GraphPad Prism 5 software (One-way ANOVA followed by Bonferroni’s multiple comparison test). **(C)** Representative Western blot of GS levels. β-actin was used for normalization. Quantification of bands was performed using the ImageJ program. Images show a representative picture from three independent experiments. Data are mean ± SE, ***p < 0.001.

The first step for the Müller cells to mount a reprogramming process requires the dedifferentiation of their phenotype by downregulating the expression of glial specific markers such as glutamine synthetase (GS). To analyze the effect of metabolic impairment on MGs’ reprogramming capacity we determined the levels of GS in MGs cultured in low (5 mM) or high (25 mM) glucose concentrations. We did not find a significant difference in the levels of this marker between these conditions (Figure 1A, left panels). Since metabolic impairment is directly associated with the production of reactive oxygen species (ROS), we studied the effect of oxidative stress induced by the exposure to oxygen peroxide (H_2_O_2_) on GS levels. Different concentrations of H_2_O_2_ were tested in order to find the optimal dose that generates oxidative damage without affecting cell viability (Supplementary Figure S1). We observed that the combination of high glucose and H_2_O_2_ (100 µM) was able to induce a significant downregulation of GS levels suggesting that this experimental condition may induce some degree of dedifferentiation (Figure 1A, lower-right panel). Western blot analysis confirmed this result (Figure 1C). This effect may be transient since GS levels showed a tend to increase when measured at longer periods of H_2_O_2_ incubation (Supplementary Figure S2).

**FIGURE 2:**
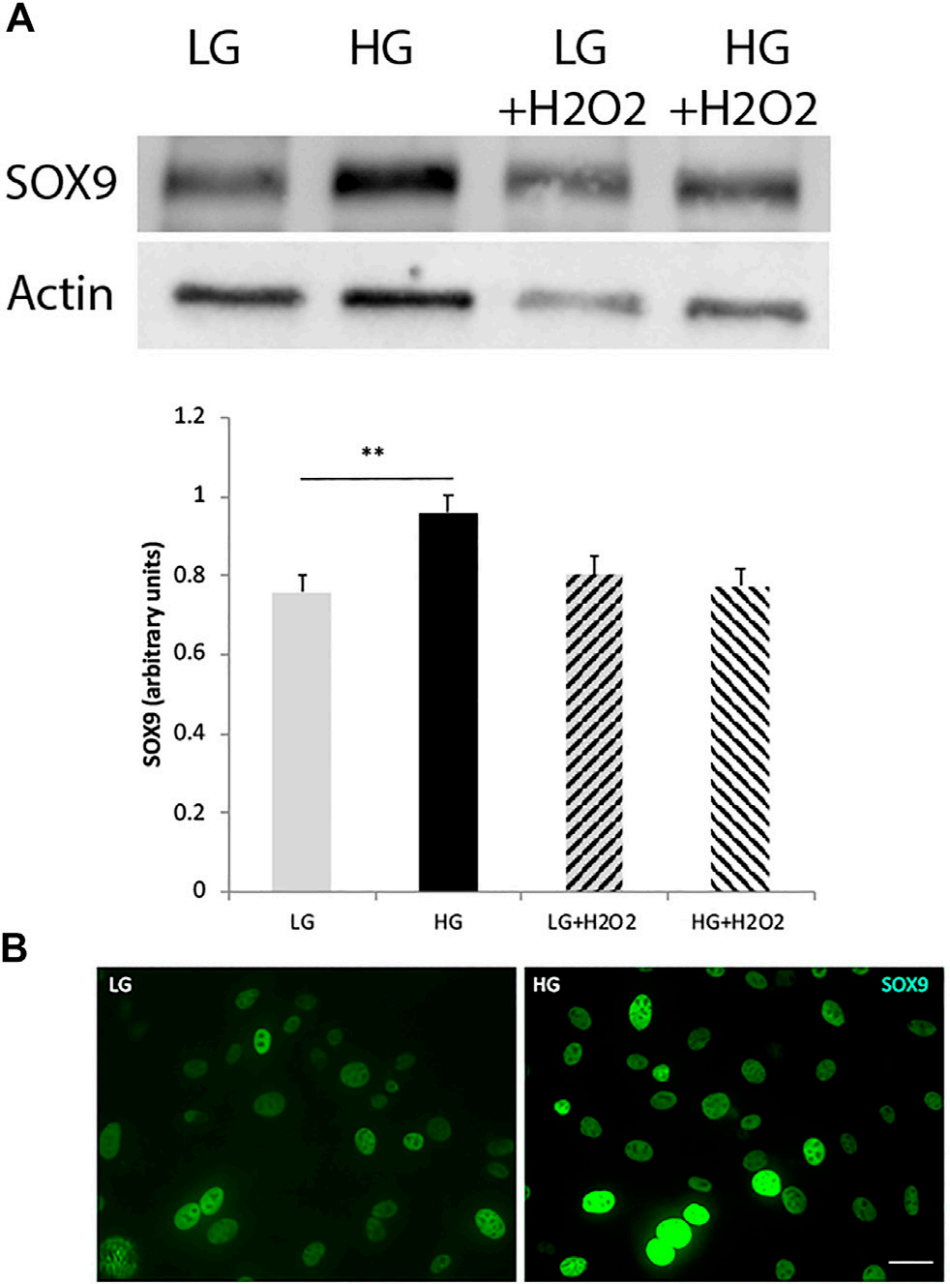
The levels of the reprogramming factor SOX9 increase under high glucose concentrations. **(A)** Western blot analysis showed an increase in SOX9 levels in MGs cultured with high glucose (HG, 25 mM), while oxidative stress (H_2_O_2_ 100 μM) had no effect on this factor’s levels. Quantification of bands was performed using the ImageJ program. β-actin was used for normalization. Data are mean ± SE of three independent experiments, **p < 0.01. **(B)** Representative immunofluorescence confirmed the increase in SOX9 levels (green). Scale bar: 20 μm. Images show a representative picture from three independent experiments.

Concomitantly to the downregulation of cell-specific markers, MGs would have to upregulate the levels of pluripotent or reprogramming factors. SOX9 is a high mobility group box transcription factor required for the establishment and maintenance of neural stem cells in both embryonic and adult CNS (Scott et al., 2010), and has been described to be expressed in adult Müller glia and retinal pigment epithelium (RPE) cells (Poché et al., 2008). We found that HG exposure was able to induce an increase in SOX9 levels in MGs, while oxidative stress, either applied individually or in combination with HG, had no effect on the levels of this factor (Figure 2A). High glucose effect on SOX9 levels was confirmed by immunofluorescence analysis (Figure 2B).

**FIGURE 3:**
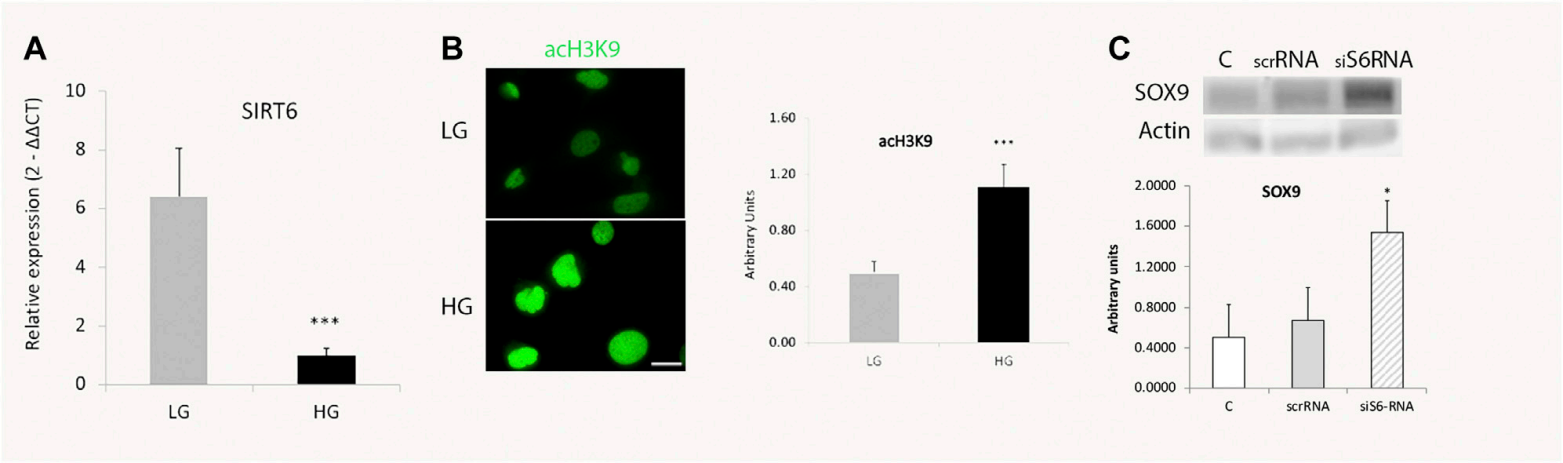
SIRT6 levels decrease in high glucose treated cells. **(A)** qPCR analysis revealed a decrease in SIRT6 mRNA levels in MGs cultured with high glucose (HG, 25 mM) compared to low glucose (LG, 5 mM). **(B)**. Representative immunofluorescence shows the acetylation levels of SIRT6 substrate H3H9 (green) in MGs cultured under low glucose (LG, 5 mM) or high glucose (HG, 25 mM). Quantification was performed using the ImageJ program. Scale bar: 10 μm. **(C)** Müller glia response to SIRT6 silencing. SIRT6 siRNA was transiently transfected and the levels of SOX9 were determined by Western blot. β-actin was used for normalization. Data are mean ± SE and images show a representative picture from three independent experiments. *p < 0.05, ***p < 0.001.

It has been demonstrated that the NAD + dependent histone deacetylase SIRT6 is a key modulator of glucose homeostasis. Moreover, several pluripotent genes like *Oct4*, *Sox2* and *Nanog* were described to be directly regulated by SIRT6 during embryonic stem cells (ESC) differentiation (Etchegaray et al., 2015). These observations prompted us to analyze SIRT6 involvement in the reprogramming capacity of MGs. Since Figure 2 showed that only glucose variations exerted a significant effect in SOX9 levels, we focused only in the effects of glucose over SIRT6 levels*.* We analyzed this parameter in MGs and observed that HG induced a decrease in SIRT6 expression levels (Figure 3A) accompanied by an increase in the acetylation status of one of its substrates (acH3K9) (Figure 3B). Remarkably, the silencing of SIRT6 by siRNA rendered an increase in SOX9 levels suggesting that this reprogramming factor could also be regulated by SIRT6 in these cells (Figure 3C).

**FIGURE 4:**
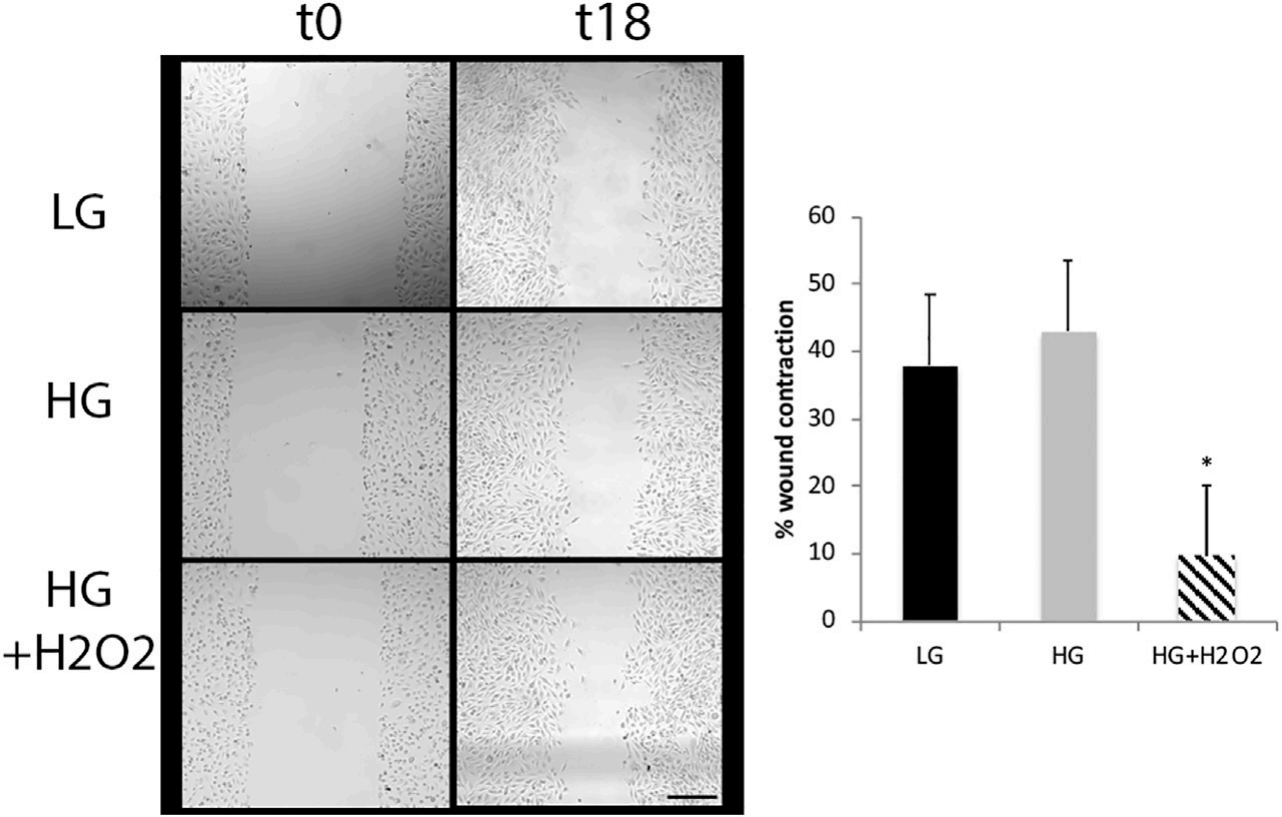
High glucose and oxidative stress induce migration of Müller cells. The wound healing assay was performed in MGs cultured under low glucose (LG, 5 mM) or high glucose (HG, 25 mM) concentrations with or without H_2_O_2_ (100 μM). Images show a representative picture from three independent experiments (three parallel scratch lesions were done in each well for each experiment) and the wound contraction was quantified as the area of the gap between wound boundaries at different time points (t0 and t18) using the ImageJ software. Data are mean ± SE, *p < 0.05. Scale bar: 200 μm.

Another step in the reprogramming process includes the migration of the cells to the layer of the damaged neurons. We tested the migration capacity of MGs by using the wound healing assay. While glucose variations did not induce a significant effect in the migration ability, the combination of HG and oxidative stress induced a decrease in the gap size between t0 and t18 indicating that these experimental conditions would favor this process (Figure 4). Oxidative stress had no significant effects under low glucose conditions (not shown).

**FIGURE 5:**
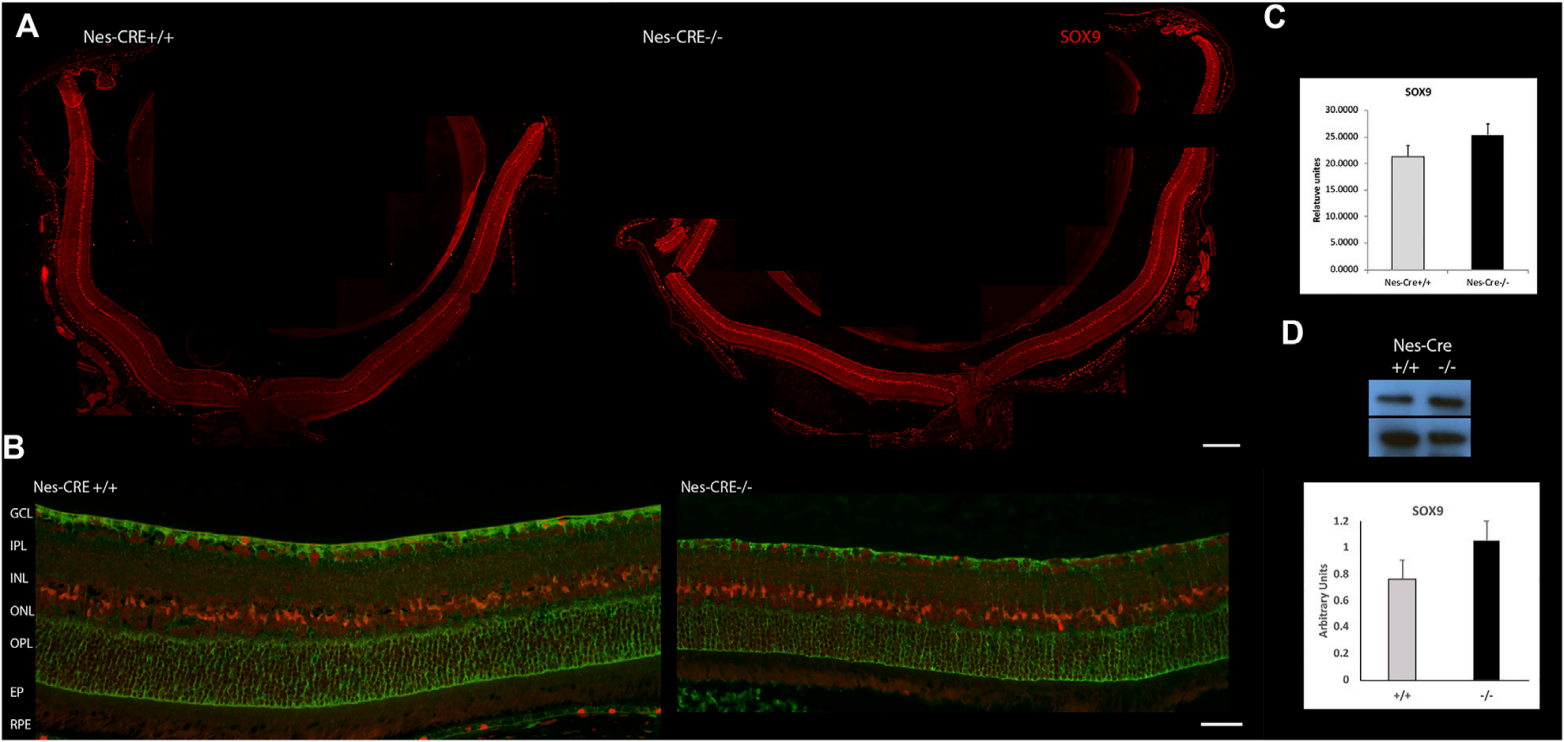
SOX9 levels in retinas from mice with a conditional deletion of SIRT6 from the CNS. **(A)** Representative immunofluorescence of SOX9 levels (red) in Nes-Cre^+/+^ (left panel, *n* = 4) and Nes-Cre^−/−^ (right panel, *n* = 5) whole retinal sections. Scale bar: 100 μm. **(B)** Higher magnification photos show colocalization of SOX9 with glutamine synthetase (green) in Müller cells and RPE. Scale bar, 50 μm Ganglion Cell Layer (GCL), Inner Plexiform Layer (IPL), Inner nuclear Layer (INL), Outer Plexiform Layer (OPL), Outer Nuclear Layer (ONL), Retinal Pigment Epithelium (RPE). **(C)** Fluorescence intensity was quantified using the ImageJ program. **(D)** Representative Western blot of SOX9 levels from retinal extracts of Nes-Cre animals. β-actin was used for normalization. Quantification of bands was performed using the ImageJ program. Data are mean ± SE.

In order to validate our *in vitro* results, we determined SOX9 levels in retinas from Nes-Cre^−/−^ mice. These animals bear a specific deletion of SIRT6 in the CNS and, hence, in their retinas (Zorrilla-Zubilete et al., 2018). Immunofluorescence studies showed that SOX9 is specifically expressed in Muller cells as it co-localizes with GS and in the retinal pigment epithelium (RPE) (Figures 5A,B). It was observed a moderate, though non-significant, increase in SOX9 levels in retinas from Nes-Cre^−/−^ mice compared to Nes-Cre^+/+^ (Figures 5C,D). This result suggests that SIRT6 regulation of SOX9 levels *in vivo* may not be direct and probably involves other factors.

**FIGURE 6:**
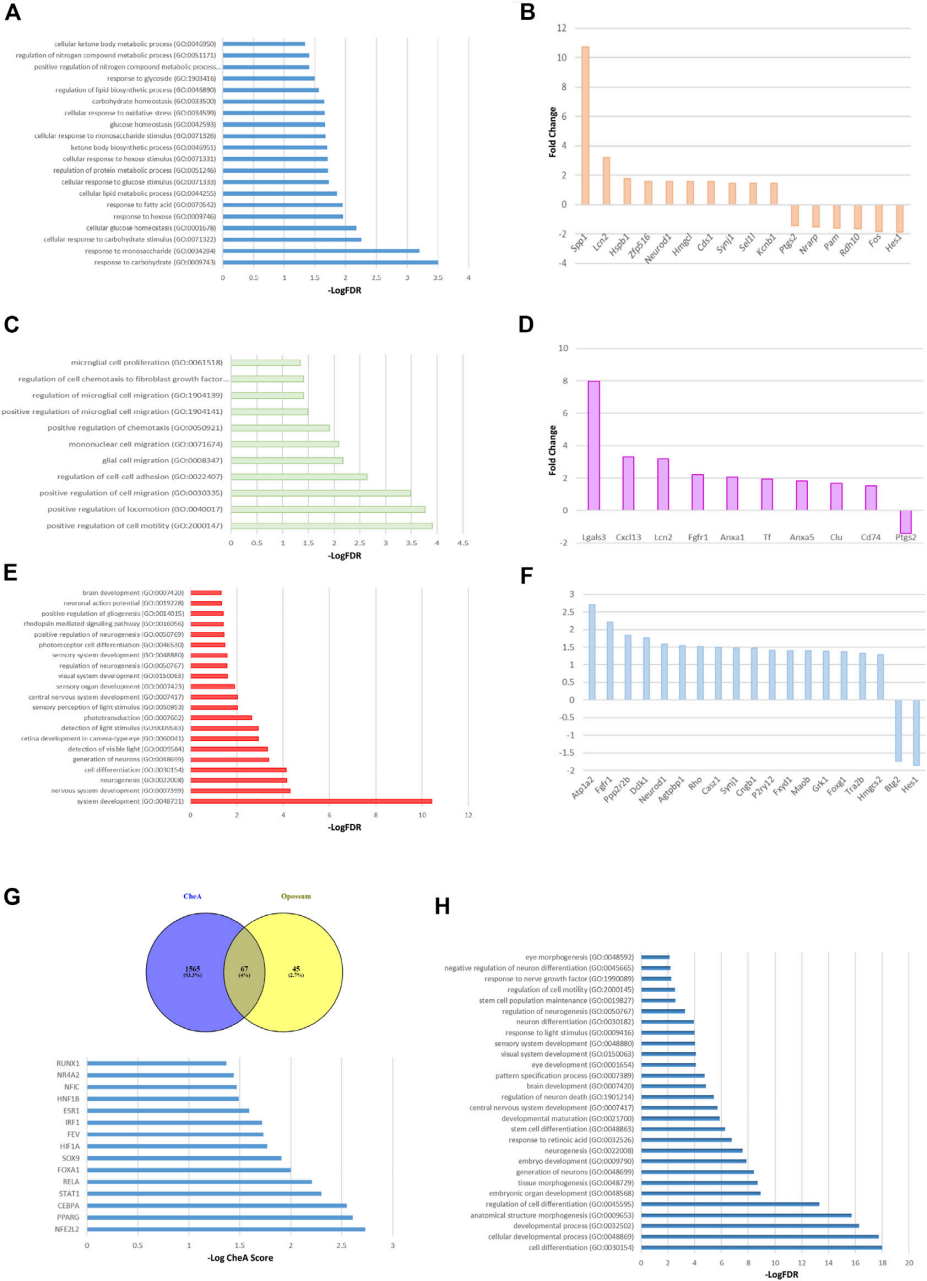
Transcriptomic analysis of Müller glial cells from a murine model of Diabetes. GO enrichment analysis of the 163 altered mRNAs shows the GO terms associated with metabolic processes **(A)**, migration and motility pathways **(C)** and cellular differentiation processes **(E)**, specially focused on neurogenesis, brain and visual sensory organs development. The top ten of upregulated and downregulated genes associated with the metabolic processes, glial cells migration and cell reprogramming are described in panels **(B, D, F)** respectively. **(G)** The top panel shows the transcription factors that could explain the changes in diabetic MGs gene expression shared between ChEA and Opossum enrichment analysis. Lower panel depicts the top 15 transcription factors, in terms of ChEA score, that explain the transcriptome of diabetic MGs. **(H)** GO analysis using the 67 predicted transcription factors. Only the GO terms associated with development, specially brain and eye development, are shown.

To deepen the *in vivo* regulatory pathways altered in Müller cells under a metabolic impaired environment, we evaluated the gene expression changes of a published dataset generated by using retinal Müller cells from diabetic rats compared to cells from healthy controls (Gerhardinger et al., 2005). Since in a diabetic state retinas are subjected to glucose variations and oxidative stress damage, among other stressors, we considered that this model would suit our experimental system. The differential expression analysis showed that under diabetic conditions, 163 genes changed their expression in Müller cells. While 146 increased their transcript levels, 17 decreased their expression levels (Supplementary Table S1). To determine the biological relevance of these transcriptional changes, we performed a Gene Ontology enrichment analysis. As expected, the transcriptomic profile showed that the enriched genes were associated with the glucose and monosacharyde metabolism, and ketone bodies synthesis and usage (Figures 6A,B; Supplementary Table S2). Interestingly, and in accordance with our *in vitro* observations, the enrichment analysis showed that metabolic impairment positively regulates several genes involved in glial cells migration (Figure 6C). This positive impact on cellular migration may mainly be due to changes in metalloproteases, gelatinases, and growth factors such as FGF (Figure 6D). Additionally, we observed a consistent enrichment in pathways associated with organogenesis, embryonic and brain development, and neurogenesis (Figure 6E). The genes mapped into developmental and neurogenesis ontologies include transcription factors such as FOXG1 and HES1, and growth factors like FGFR and NeuroD1 (Figure 6F). To validate these results, the GO enrichment was corroborated with the Reactome database obtaining similar results (Supplementary Table S3). Upregulated genes were referenced to the KEGG pathways database and remarkably, we found that many functional categories affected in cells from diabetic animals were directly related to SIRT6 function like glycolysis, Hif1α pathway and phototransduction (Supplementary Table S4). Lastly, to determine which transcription factors (TF) could be responsible for the transcriptional changes observed in MGs from diabetic rats, we used two approaches to perform TF enrichment analysis. We used the chromatin enrichment (ChEA) algorithm that includes results of ENCODE chromatin immunoprecipitation (ChIP), and the Opossum 3.0 algorithm that predicts TF binding sites. Figure 6G shows that 67 TFs are shared between ChEA and Opossum enrichment analysis. Interestingly, we found SOX9 among the top 10 transcription factors in terms of ChEA score, confirming that this TF may have a key role in regulating the differential expression program observed in Müller cells under metabolic impaired conditions (Figure 6G; Supplementary Tables S5–S7). Moreover, the GO enrichment analysis of these predicted 67 TFs showed that these factors are enriched in biological processes like cell differentiation and development (including brain and eye development) as well as neurogenesis (Figure 6H; Supplementary Table S7). Likewise, we performed an enrichment analysis using the list of TFs generated by the ChEA algorithm and validated with Reactome, and observed several TFs that may be involved in regulating the acquisition of a pluripotent phenotype like KLF4, NANOG, SOX2, POU5F1 and LIN28 (Supplementary Figure S3), which are also regulated by SIRT6 (Etchegaray et al., 2015; Kugel et al., 2016). Further studies are needed in order to elucidate the role of other pathways that may have opposite effects *in vivo,* thus preventing MGs to adopt a fully reprogramming phenotype in physiological conditions.

## Discussion

Increasing evidence suggests that metabolic alterations strongly influence the initiation and progression of neurodegenerative disorders including retinopathies (Lin and Beal, 2006; de la Montea and Tongd, 2014; Kim et al., 2017; Harder et al., 2020). Cells of the CNS that do not cope properly with the metabolic, morphological and neurophysiological changes needed to maintain the homeostasis of the tissue would eventually contribute to the development of neurodegenerative processes. As a highly metabolic tissue, the retina needs to adapt as well. Müller cells, the major glial component of the retina, play a critical role in retinal metabolism and are among the first cells to demonstrate metabolic changes in retinal stress or disease. They are under the focus of current investigations due to their potential regeneration capacity. Adult Müller glial cells retain a progenitor cell-like gene expression profile that makes them a suitable source of regenerative cells (Das et al., 2006). Since the availability of glucose and oxygen are important regulatory elements that may help directing stem cell fate, in this work we aimed to study the effect of glucose variations and oxidative stress in the reprogramming capacity of Müller cells and tried to identify the epigenetic mechanisms that regulate this process by studying the role of the histone deacetylase SIRT6.

In order for MGs to initiate a reprogramming process they have to adopt a dedifferentiated phenotype by downregulating their specific glial markers such as glutamine synthetase (GS). We analyzed GS levels in MGs cultured under different metabolic settings and found that the combination of high glucose and oxidative stress was able to induce a significant decrease in GS levels suggesting that this experimental condition may cause some degree of dedifferentiation. The balance between pluripotent embryonic and multipotent adult stem cells may be related to metabolism variations which, in turn, influence the cellular redox state. In this sense, changes in the levels of reactive oxygen species (ROS) could mediate the interplay between metabolism and stem cell fate (Ryu et al., 2015). Our observations are in accordance with the fact that optimal ROS levels are required for the onset of a reprogramming process (Zhou et al., 2016). Moreover, GS levels variations may explain the reduced dependence on exogenous glutamine, which is a feature of pluripotent stem cells (Vardhana et al., 2019). However, since GS downregulation may reduce the protection against glutamate excitotoxicity, this decrease should be temporary to avoid further damage of the tissue under a harmful stimulus.

In parallel with the downregulation of specific markers, MGs would have to upregulate the expression of pluripotent or reprogramming factors. The high mobility group box transcription factor (TF) SOX9 was demonstrated to be indispensable for the establishment and maintenance of neural stem cells in both embryonic and adult CNS (Scott et al., 2010). It was described to be vital in triggering the switch from the neurogenic to the gliogenic program at the germinal zones of different neural tissues. During retinogenesis, Sox9 is expressed in multipotent progenitor cells, although in the adult retina it is expressed only in Müller glia and RPE cells (Poché et al., 2008). We determined that high glucose induced an increase in SOX9 levels in MGs; while oxidative stress, applied individually or in combination with HG, had no effect on the levels of this factor. Zhou et al. showed that the addition of exogenous hydrogen peroxide induced a biphasic pattern of mouse embryonic fibroblasts (MEFs) reprogramming capacity. They observed a tendency towards an increase in the yield of induced pluripotent stem cells (iPSC) derived from MEFs treated with a low dose of H_2_O_2_, as well as an impaired reprogramming efficiency with higher doses (Zhou et al., 2016). Further studies using different incubation times with H_2_O_2_ will help us define whether a transient acquisition of a pluripotent phenotype could be achieved *in vitro*.

The NAD^+^-dependent histone deacetylase SIRT6 has been described as a critical modulator of metabolism by repressing the expression of several glycolytic genes (Zhong et al., 2010) and as a key regulator of the retinal function (Silberman et al., 2014). We determined that HG was able to induce a decrease in SIRT6 levels and an increase in acH3K9 levels in Müller cells, as seen previously in primary cell cultures (Zorrilla-Zubilete et al., 2018). Since glycolytic enzymes represent candidate targets for enhancing somatic reprogramming, inducing the expression and/or activity of glycolytic enzymes in Müller cells would improve their reprogramming capacity. Remarkably, SIRT6 was described to regulate embryonic stem cell differentiation by modulating the acetylation status of its substrates at the promoter level of several pluripotent genes like *sox2*, *nanog* and *oct4* (Etchegaray et al., 2015). In this sense, we found that the downregulation of SIRT6 expression by siRNA rendered an increase in SOX9 levels as seen in HG-treated cells. Considering that stem cells have been described to prefer aerobic glycolysis rather than oxidative phosphorylation (Bigarella et al., 2014; Zheng et al., 2016), and that an increment in ROS was shown to be required for the onset of a reprogramming process, the modulation of the shift in the metabolic imbalance between these two processes may play a critical role in defining whether cells are in quiescent, pluripotent, proliferative, or differentiating states (Shyh-Chang et al., 2013). The downregulation of SIRT6 levels by the exposure to high glucose might induce the expression and activity of glycolytic enzymes and the expression of pluripotent genes enhancing the reprogramming capacity of MGs thus facilitating a regenerative response. This observation is in accordance with recent works that show that a histone deacetylase inhibitor promotes chromatin accessibility at key gene loci in MGs allowing a more effective reprogramming process (Jorstad et al., 2017), and that several pluripotent transcriptional factors are increased in SIRT6-knockout MEFs (Xu et al., 2019). Additional studies will help us define the role of SIRT6 in MG’s reprogramming capacity. It is worth noting that metabolic impairment may induce an increased burden of DNA damage causing cells to accumulate genomic instability. The concentration of H_2_O_2_ used in our *in vitro* experiments was able to induce DNA damage as seen by the increase in ɣH2AX levels depicted in Supplementary Figure S1. SIRT6 promotes DNA double-strand break (DSB) repair contributing to the maintenance of genome stability (Toiber et al., 2013; Onn et al., 2020). Thus, if DNA repair becomes a priority under oxidative stress conditions, a redistribution of SIRT6 to sites of breaks, and the consequent reduction of its levels or activity in other genome locations, may lead to transcriptional misregulation favoring metabolic imbalance and neurodegeneration. Moreover, DNA damage can activate the poly ADP-ribose polymerase (PARP1) that catabolizes NAD+, and the extent of NAD+ depletion may likely correlate with the extent of the damage. Several studies have shown that the effect of H_2_O_2_ on NAD+ levels depends on the cell type and the concentration of peroxide used (Zhu et al., 2016; Ye et al., 2019; Martín-Guerrero et al., 2020). Additional studies are needed in order to determine if NAD+ levels are affected in our system, and if so, whether some of the peroxide effects could be reversed by using a NAD+ precursor.

Mice with a conditional deletion of SIRT6 in the CNS (Nes-Cre^−/−^) have been shown to exhibit a neurodegenerative phenotype, including behavior impairment, learning and memory deficit. They also showed neurodegenerative markers like increased DNA damage and apoptotic cells, and augmented Tau phosphorylation in the brain (Kaluski et al., 2017; Portillo et al., 2021). Moreover, retinas from these mice presented structural and molecular evidence of neurodegeneration (Zorrilla-Zubilete et al., 2018). We found that retinas from Nes-Cre^−/−^ mice showed a moderate, yet non-significant, increase in SOX9 levels compared to Nes-Cre^+/+^ suggesting that *in vivo* regulation of this factor by SIRT6 may be more variable and may involve multiple players. It is worth mentioning that not all Müller cells of a retina may respond to a pathogenic stimulus in the same way (Bringmann et al., 2009). Such a heterogeneity between neighboring Müller cells in the same region of the retina would explain the lack of significance of SOX9 levels observed in the tissue and reveal how challenging it could be to identify *in vivo* the subpopulation/s of these cells with reprogramming capacity.

Another key step during reprogramming involves the migration of undifferentiated cells to the site of damage. We tested the migration capacity of MGs and found that, while glucose variations did not induce a significant effect, the combination of HG and oxidative stress stimulates this process. This result differs from previous observations in retinal astrocytes (Shin et al., 2014) and other cell types (Lamers et al., 2011). However, activated microglia results in increased proliferation and migration as described in diabetic retinopathy, a disease that is a direct consequence of sustained hyperglycemia and oxidative stress induced during Diabetes (Altmann and Schmidt, 2018). Thus, further studies will be necessary to define the signals that modulate the migration capacity of retinal Müller cells during a reprogramming process which may differ from the ones involved in a pathological disorder.

In an attempt to better understand the regulatory pathways altered in Müller cells under a metabolic impaired environment like the one in a diabetic state, we evaluated the gene expression profile of Müller cells from a published dataset generated by using murine a model of Diabetes compared to cells from healthy controls (Gerhardinger et al., 2005). The differential expression analysis showed 146 upregulated transcripts and 17 downregulated transcripts under diabetic conditions. To determine the biological relevance of these transcriptional changes, we did a Gene Onthology enrichment analysis that revealed, as expected, that the transcriptomic change was enriched in genes associated with glucose and monosacharyde metabolism, as well as ketone bodies synthesis and usage. Interestingly, some of the observed changes were also implicated in lipid metabolism, mainly due to the change in the expression of the transcription factors PPARα and NF-κβ, and the expression of glycolytic regulatory enzymes HK2 and PFKFB2. Moreover, in accordance with our *in vitro* observations, the enrichment analysis also showed that a diabetic state positively regulates genes related to migration processes. This positive impact on cellular migration may be due to different genes that code for metalloproteases, gelatinases, and growth factors such as FGF. Furthermore, we observed a consistent enrichment in pathways associated with organogenesis, embryonic and brain development, and neurogenesis. Interestingly, when upregulated genes were referenced to the KEGG pathways database we found that many functional categories affected in MGs from diabetic animals were directly related to SIRT6 function like glycolysis, Hif1α pathway and phototransduction. These observations support previous findings from our group and others, further endorsing the role of SIRT6 as a key modulator of glucose homeostasis and retinal function (Zhong et al., 2010; Silberman et al., 2014).

To determine which transcription factors could be responsible for the transcriptional changes observed in diabetic Müller glial cells, we performed two TF enrichment analysis. We used a ChEA algorithm that includes the ENCODE ChIP results in its analysis and the Opossum 3.0 algorithm that predicts putative TF binding sites. When we crossed the results from both analyses, we obtained a list of 67 shared transcription factors, which includes SOX9 among the top 10 factors, confirming that this TF may play a strategic role in regulating the differential expression program observed in Müller cells under metabolic impaired conditions. The GO enrichment analysis of these predicted 67 TFs showed that these factors are enriched in biological processes like cell differentiation and development (including brain, visual systems and eye development) as well as neurogenesis and stem cell differentiation. Additionally, we used the list of TFs generated by the ChEA algorithm to perform an enrichment analysis and found several factors that could be involved in the acquisition of a pluripotent phenotype like KLF4, LIN28, SOX2, NANOG and POU5F1 (Supplementary Figure S3). It is worth noting that these TFs were described to be regulated by SIRT6 (Etchegaray et al., 2015; Kugel et al., 2016) further validating the role of this enzyme in regulating reprogramming processes and modulating stem cells’ fate during development.

Our results suggest that metabolic impaired Müller glial cells could alter their genetic landscape favoring a phenotype associated with a developmental profile that shares some characteristics with a reprogramming state. Müller cells response to a metabolic defy *in vivo* may involve other regulatory pathways that exert opposite inhibitory effects preventing MGs to adopt a fully reprogramming phenotype in physiological conditions. This complexity exposes the need of additional studies that will help us understand the mechanisms that regulate MGs response under metabolic impaired conditions. This, in turn, would help develop new therapeutic strategies to treat neurodegenerative diseases.

## Data Availability

The original contributions presented in the study are included in the article/Supplementary Material, further inquiries can be directed to the corresponding author.

## Supplementary Material

Supplementary Figure 1 Oxidative stress induced by H_2_O_2_ exposure. Several concentrations of H_2_O_2_ (50 µM, 100 µM, 500 µM) were used to determine the optimal dose that does not affect cell viability. Immunofluorescence depicts DNA damage as measured by gHAX (green) foci formation. Images show a representative picture from three independent experiments. Quantification was performed using the ImageJ program. **p* < 0.05. Magnification 10× and 40×.

Supplementary Figure 2 Temporary effect of oxidative stress on glutamine synthetase (GS) levels. Western blot analysis showed that while a 2 h incubation of MGs with H_2_O_2_ (100 mM) induces a decrease in GS levels, a 24 h incubation partly reverts this effect. Quantification of bands was performed using the ImageJ program. b-actin was used for normalization. Data are mean ±SE of three independent experiments, ***p* < 0.01.

Supplementary Figure 3 Enrichment analysis of the putative regulatory transcription factors from ChEA.

Supplementary Table 1 Differential expression analysis.

Supplementary Table 2 GO Enrichment analysis.

Supplementary Table 3 Reactome.

Supplementary Table 4 KEGG pathways.

Supplementary Table 5 Chromatin Enrichment analysis (ChEA).

Supplementary Table 6 Opposum analysis.

Supplementary Table 7 GO Enrichment analysis of putative TFs.
