# Supplementary figures and images for: Metabolic Imbalance Effect on Retinal Müller Glial Cells Reprogramming Capacity: Involvement of Histone Deacetylase SIRT6
Source: Front Genet. 2021 Nov 4;12:769723. doi: 10.3389/fgene.2021.769723 (PMC8599966; doi:10.3389/fgene.2021.769723)

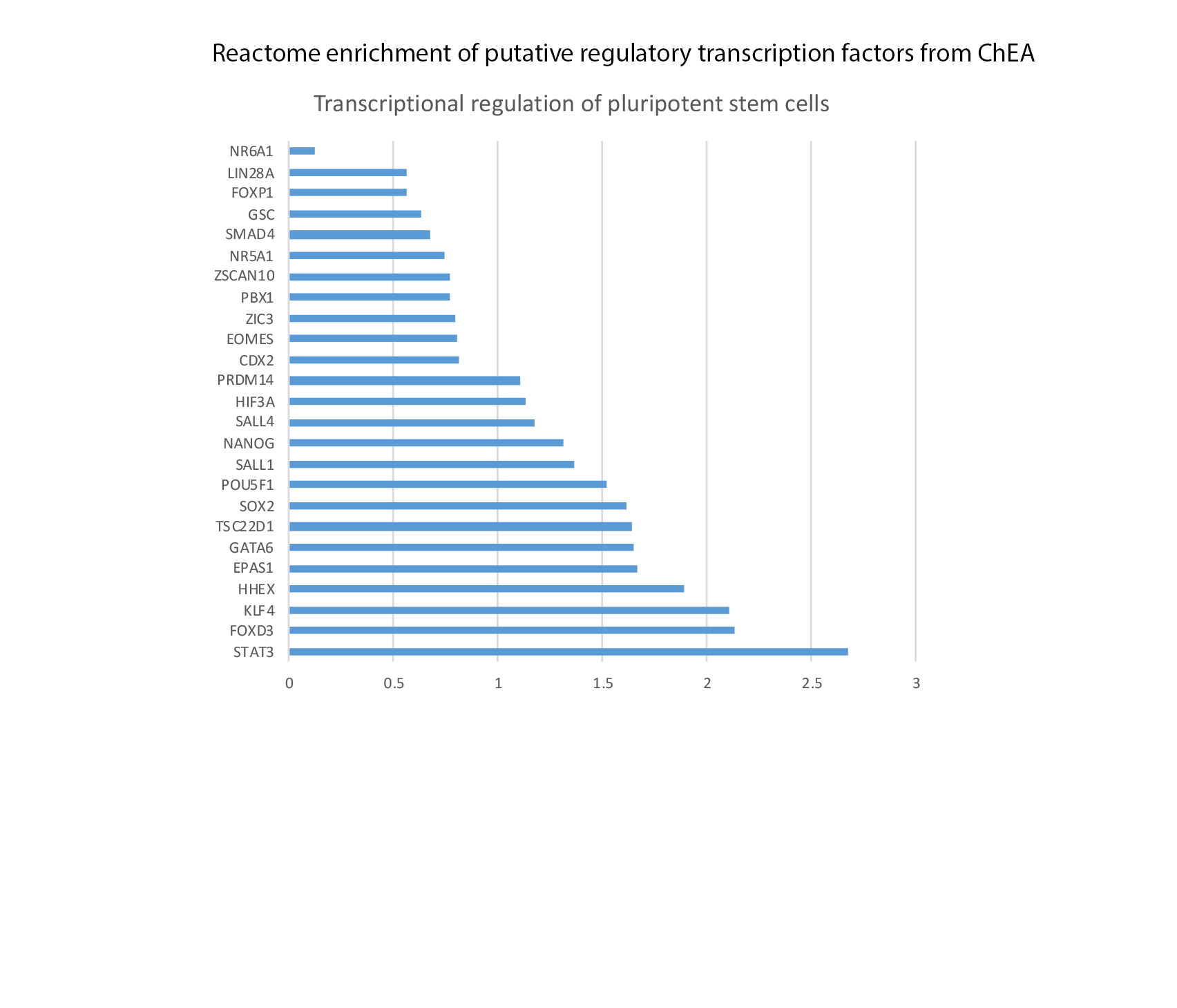

Supplement: Supplementary file 1 [file Image3.TIF]

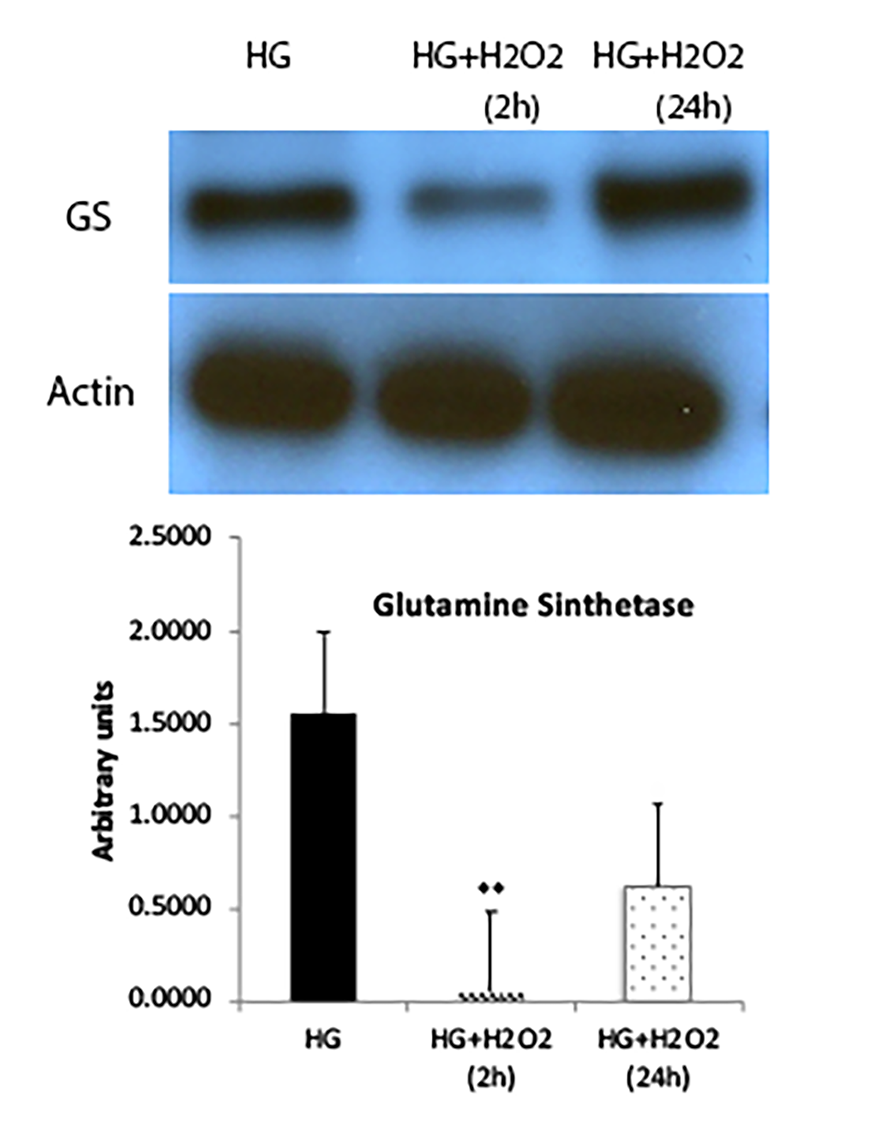

Supplement: Supplementary file 2 [file Image2.TIF]

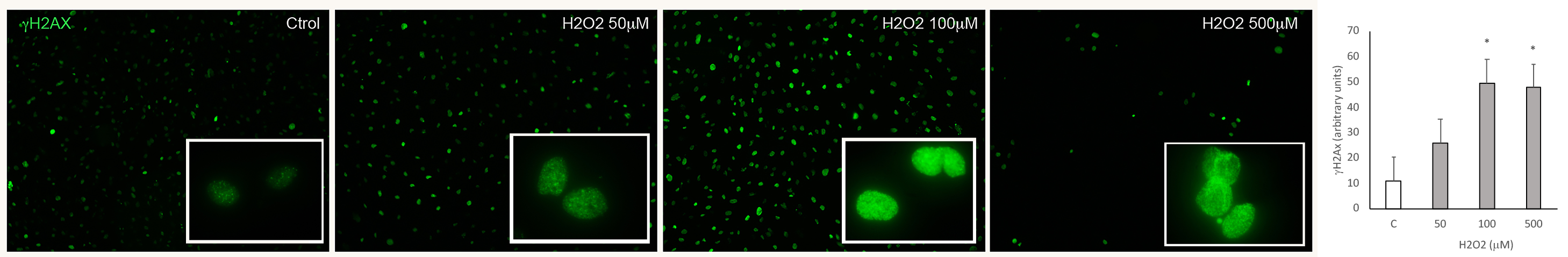

Supplement: Supplementary file 3 [file Image1.TIF]
